# Supplementary material for: Neural Differentiation Potential of Mesenchymal Stem Cells Enhanced by Biocompatible Chitosan-Gold Nanocomposites
Source: Cells. 2022 Jun 7;11(12):1861. doi: 10.3390/cells11121861 (PMC9221394; doi:10.3390/cells11121861)
Supplement: Supplementary file 1 [file cells-11-01861-s001.zip › cells-1742602-supplementary.pdf]

Neural Differentiation Potential of Mesenchymal Stem Cells enhanced by Biocompatible Chitosan-Gold Nanocomposites

Supplementary data

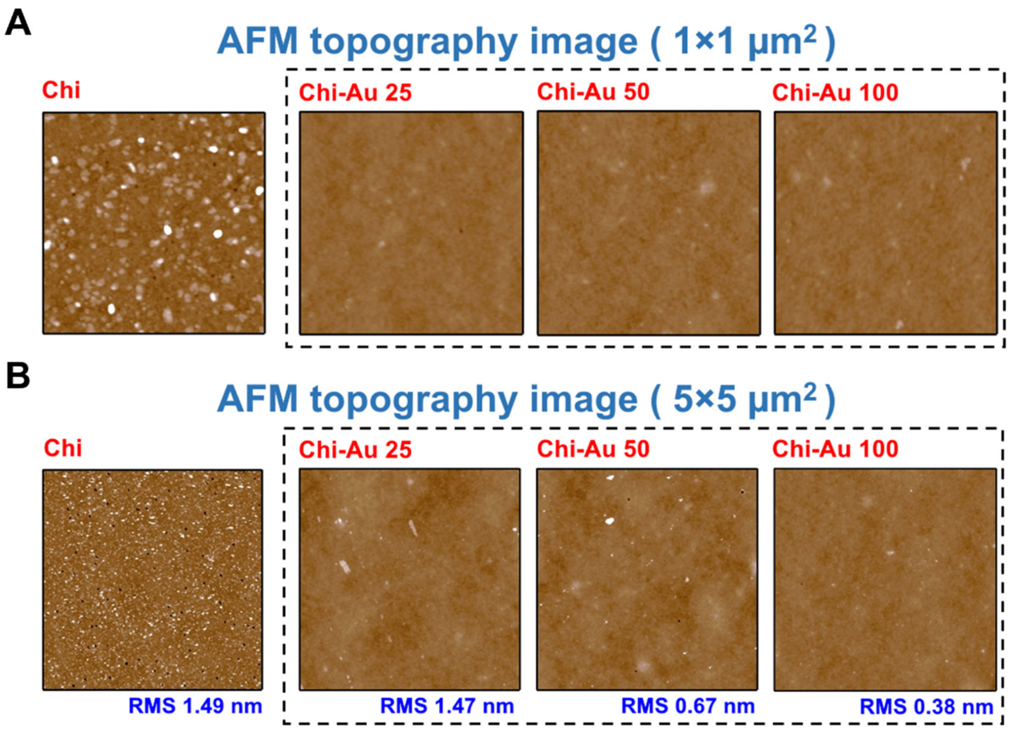

Figure S1. Surface topography of Chi combined with Au nanoparticles observed by AFM. Pure Chi, Chi-Au 25 ppm, Chi-Au 50 ppm, and Chi-Au 100 ppm were observed at (**A**) 1×1 μm^2 and (**B**) 5×5 μm^2. The values of root mean square (RMS) for pure Chi, Chi-Au 25 ppm, Chi-Au 50 ppm, and Chi-Au 100 ppm were 1.49 nm, 1.47 nm, 0.67 nm, and 0.38 nm, respectively. The results represent one of three independent experiments.

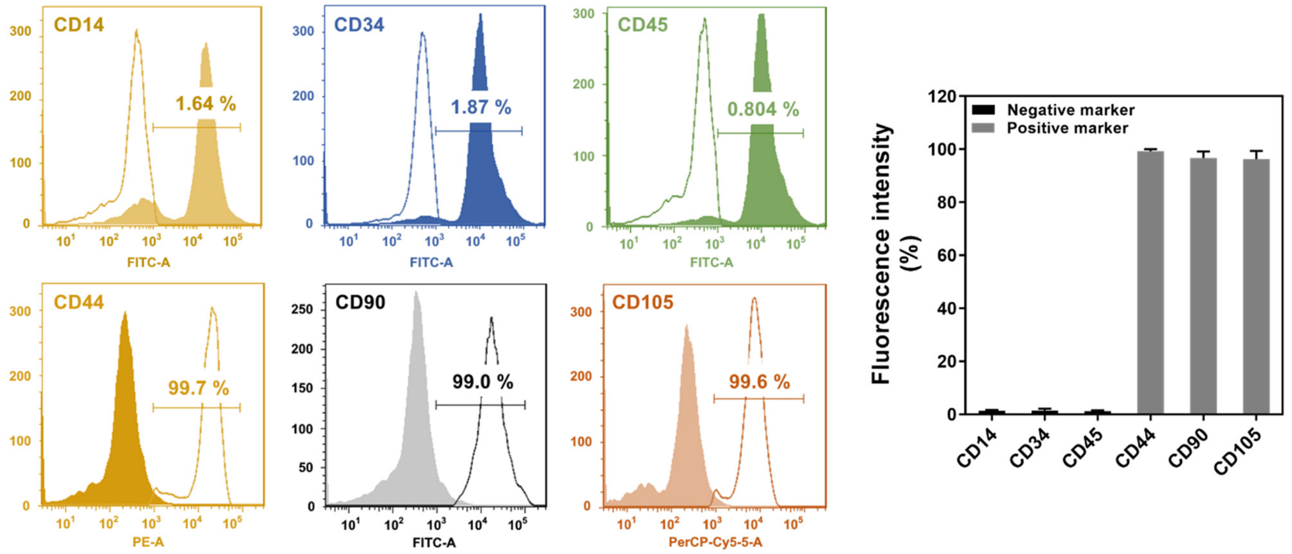

Figure S2. Phenotype characterization of MSCs. The specific markers expressed in MSCs were identified by flow cytometry. The expression of endothelial markers, CD14, CD34, and CD45 were quantified as 1%, indicating negative markers. The phenotypes of positive markers in human MSCs, CD44, CD90, and CD105 were expressed as 99%, 97%, and 96%, respectively. The results represent one of three independent experiments.

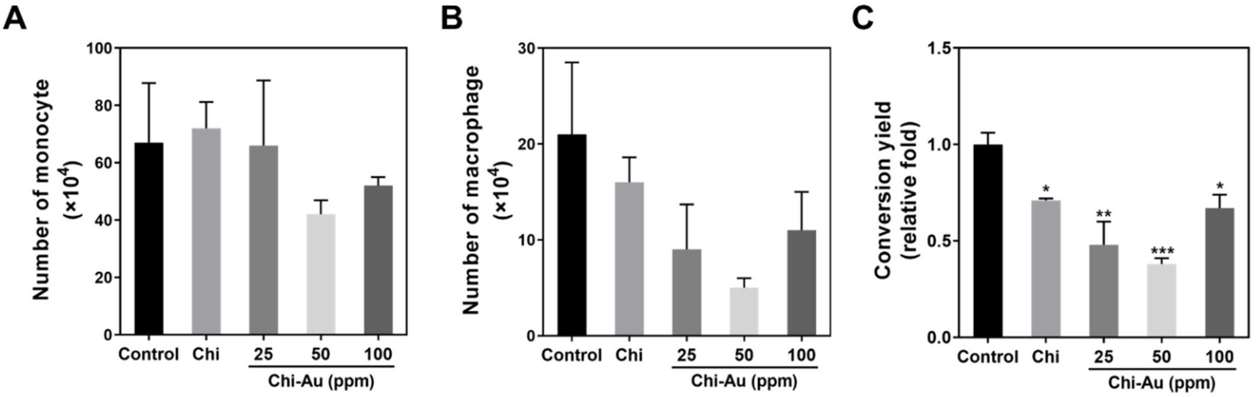

Figure S3. Monocyte-macrophage conversion yield seeded on various Chi-Au nanocomposites at 96 hours. (**A**) Quantification of number of monocytes. (**B**) Quantification of number of macrophages. (**C**) The conversion of monocytes into macrophages was semi-quantified. The results demonstrated the monocyte conversion yield was the lowest in the Chi-Au 50 ppm group, indicating Chi-Au 50 ppm showed better biocompatibility among the different nanocomposites. Data represent one of three independent experiments. *$p < 0.05$, **$p < 0.01$, ***$p < 0.001$: compared to the Control (TCPS).

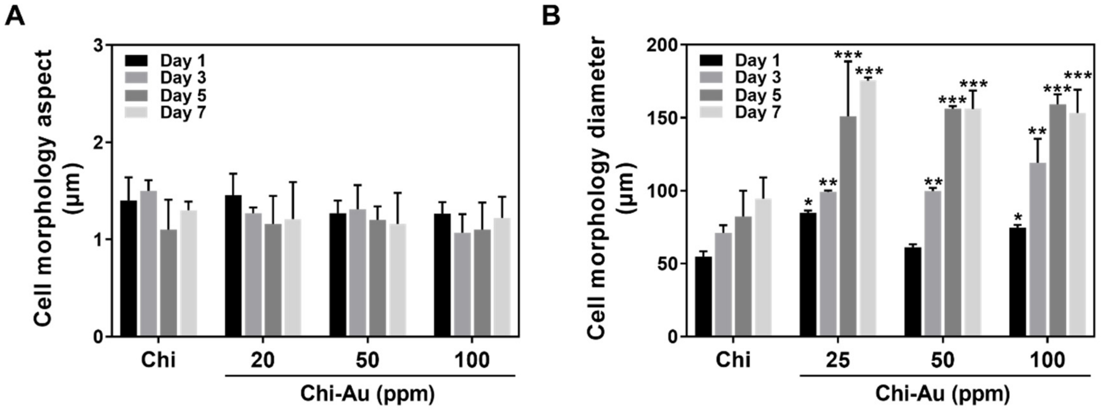

Figure S4. Quantification of cell morphology (**A**) aspect and (**B**) diameter for MSCs seeded on Chi-Au nanocomposites after 1, 3, 5, and 7 days. Data are presented as the mean ± SD (n = 3). **p* < 0.05, ***p* < 0.01, ****p* < 0.001: compared to the Control (TCPS).

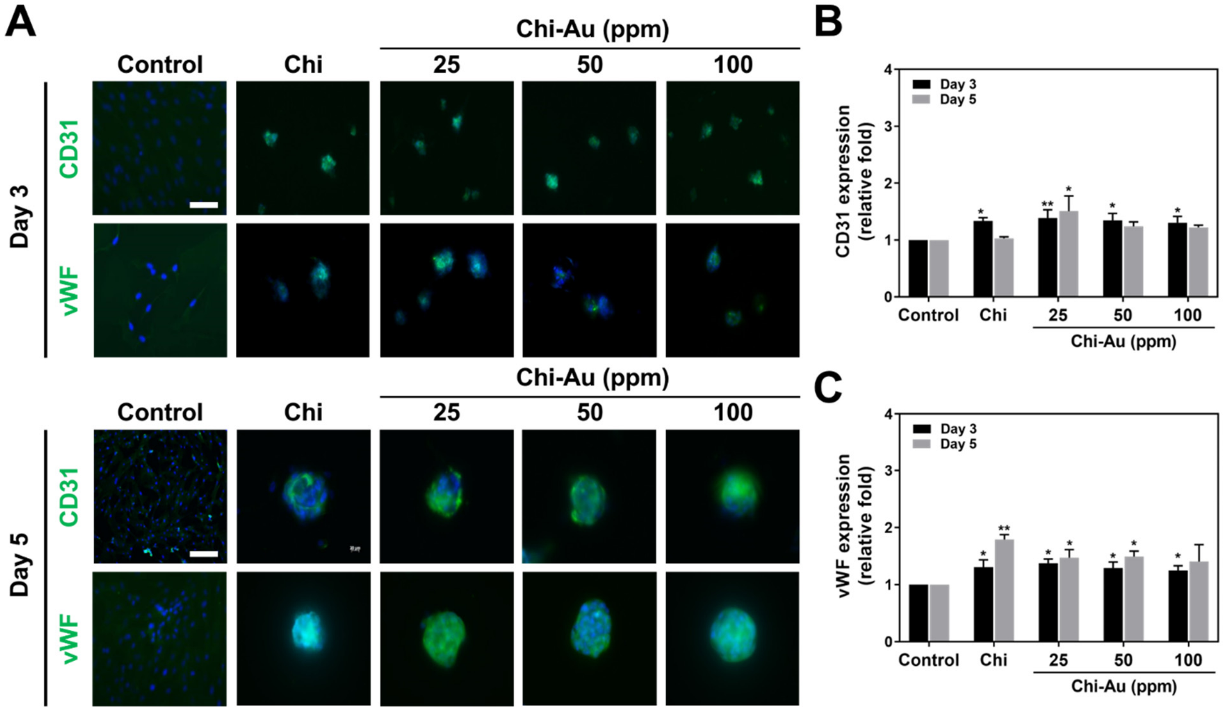

Figure S5. Endothelial differentiation capacities of MSCs induced by Chi-Au nanocomposites were measured by immunofluorescence staining at 3 and 5 days. The MSCs were first stained with primary antibodies (CD31 and vWF) and with FITC immunoglobulin second antibodies conjugation (green fluorescence). Cell nuclei were detected by DAPI (blue fluorescence). (**A**) Images of endothelialization markers (CD31 and vWF) expression are depicted. (**B**,**C**) Expression of endothelialization markers at 3 and 5 days were semi-quantified. Scale bar was set as 100 μm. Results are presented as mean ± SD (n = 3). *$p < 0.05$, **$p < 0.01$: compared to the Control (TCPS).
